# Sonographic Assessment of Splenic Manifestations in Sickle Cell Disease Patients and Its Relation to Hematological Parameters: A Cross-Sectional Study in Basra, Iraq

**DOI:** 10.7759/cureus.72322

**Published:** 2024-10-24

**Authors:** Hussein S Sadeq, Amenah S Abdulkareem, Qutaiba M Dawood, Asaad A Khalaf

**Affiliations:** 1 Department of Radiology, Basra Teaching Hospital, Basra Health Directorate, Basra, IRQ; 2 Department of Internal Medicine, Al Zahraa College of Medicine, University of Basra, Basra, IRQ; 3 Basra Hematology Center, Basra Health Directorate, Basra, IRQ

**Keywords:** autosplenectomy, gamna gandy bodies, sickle cell disease (scd), spleen infarction, spleen size, ultrasound (u/s)

## Abstract

Background

Hemoglobinopathies, such as sickle cell disease (SCD), are inherited disorders of hemoglobin (Hb) synthesis. SCD presents with complex clinical manifestations, including anemia, painful episodes, and organ damage due to recurrent vaso-occlusion. The spleen is one of the first organs affected in SCD patients, with hyposplenism typically occurring by age five. However, older patients may show variations in splenic size, which can complicate the diagnosis.

Objective

This study aimed to investigate the radiological manifestations of the spleen in patients with SCD using sonographic assessment and the relationship between these manifestations and the patient's clinical and laboratory findings.

Methodology

This descriptive cross-sectional study was carried out at the Basra Hematology Center, which is the largest center for hematological diseases in the southern part of Iraq. It was carried out over a three-month period from May 22 to August 22, 2024. A total of 81 patients aged 15 and above, diagnosed with SCD, and attending the outpatient clinic at Basra Hematology Center in Basra city were included. Participants were identified through medical records and referrals from private outpatient clinics. Inclusion criteria ensured participants had a confirmed SCD diagnosis and met the age requirements. Exclusion criteria eliminated individuals with sickle cell trait, other hemolytic anemias, leukemia, relevant co-morbidities, or those who declined participation. After obtaining informed consent, each patient was interviewed by the research team, their blood was taken for lab tests, and then an ultrasound scan of their spleen was performed.

Results

Regarding the demographic characteristics of patients, age showed a statistically significant difference across the groups (p=0.017), indicating that marked splenomegaly is associated with younger individuals (mean age of 19.50 ± 4.95 years) compared to autosplenectomy, which is linked to older patients (mean age of 35.42 ± 11.84 years). Regarding radiological findings, splenic size was categorized into normal size, splenomegaly, small spleen, and autosplenectomy. Increased echogenicity is more frequently seen in the small spleen group (87.5%, p<0.001). Focal lesions, such as infarctions, were relatively uncommon and did not show significant variation across the groups. However, the presence of Gamna-Gandy bodies (GGB) was reported in a few patients and did not vary significantly among spleen status groups. The laboratory data showed notable significant differences in several key blood parameters. Hb levels were significantly lower in patients with marked splenomegaly (6.65 ± 0.21 g/dl) compared to other groups (p=0.01). White blood cell count (WBC) and platelet count (PLT) were significantly higher in the autosplenectomy group (WBC 12.29 ± 5.67 x 10³/μL; PLT 426.16 ± 222.85 x 10³/μL), both with p-values <0.001.

Conclusion

This study examined splenic changes in SCD patients, finding that autosplenectomy is more common in older patients, while splenomegaly is prevalent in younger ones. Increased splenic echogenicity indicated fibrosis, and patients with splenomegaly showed lower hemoglobin levels. Autosplenectomy was associated with higher WBC and PLT levels. Although elevated HbF and certain genetic factors seemed protective against splenic atrophy, these findings were not statistically significant, warranting further research.

## Introduction

Hemoglobinopathies are inherited disorders of hemoglobin (Hb) synthesis, including sickle cell disease (SCD), which is the most prevalent monogenic disorder globally [1,2]. SCD is a group of inherited hemoglobinopathies characterized by mutations affecting the β-globin chain of hemoglobin. This mutation can result in an abnormal form of Hb called hemoglobin S (HbS) [1].

HbS arises from substituting glutamic acid with valine at the sixth position of the β-globin chain within hemoglobin. Severe manifestations of SCD encompass hemoglobin SS, which results from homozygous inheritance of HbS, and S/β0 thalassemia, arising from the co-inheritance of HbS with the β0 thalassemia mutation. Additionally, other forms of SCD emerge when HbS coexists with other β-globin gene mutations, such as hemoglobin C, hemoglobin D-Los Angeles/Punjab, or β+ thalassemia [3].

Each year, approximately 300,000 infants worldwide are born with SCD [1]. In 2015, the total number of SCD patients in Iraq was 5,124. The prevalence of SCD increased slightly from 13.1 per 100,000 in 2010 to 13.9 per 100,000 in 2015, while the incidence decreased from 19.7 per 100,000 in 2010 to 13.2 per 100,000 in 2015. The highest prevalence was recorded in Basra province (124 per 100,000), the southernmost province of Iraq, and the lowest was recorded in Sulaymaniyah and Salahaldin (0.3 per 100,000). The male-to-female ratio was 1.1:1 [4].

Individuals with SCD frequently encounter multiple types of sickle cell crises [5]. The mechanisms underlying SCD manifestations are complex and multifactorial [6]. Microvascular obstruction caused by abnormally shaped erythrocytes results in painful tissue ischemia, and the subsequent restoration of blood flow causes reperfusion injury [7,8]. This recurring series of events leads to progressive ischemic organ damage and chronic complications, which characterize SCD [1].

SCD clinical manifestations include anemia, susceptibility to infections, and acute painful episodes, which are the hallmarks of SCD. Consequently, this condition can lead to other complications such as stroke, acute chest syndrome, priapism, leg ulceration, and chronic organ failure [9].

The spleen is the initial organ affected, and evidence of hyposplenism often emerges in most children before 12 months of age [10,11]. During the neonatal period, the spleen in sickle cell anemia (SCA) appears morphologically and functionally normal. However, over time, progressive injury occurs due to the Hb switch [10]. Gamna-Gandy bodies (GGBs) are granuloma-like nodules characteristic in SCD patients' spleen. These structures arise due to periarteriolar hemorrhage followed by fibrosis and the deposition of iron pigments in the spleen. However, the true significance of GGBs is still unknown [12]. The precise sequence of pathogenic events leading to splenic alterations in SCA patients remains hypothetical. It is postulated to involve a randomly occurring sequence of vaso-occlusion, followed by ischemia, leading to progressive fibrosis and atrophy. Ultimately, this process results in what is commonly called autosplenectomy. Typically, this process is completed by age five in individuals with SCA [10].

However, normal splenic size and splenomegaly can be observed in older SCA patients. This poses challenges in interpretation due to potential interference from genetic or infectious factors. Notably, persistently elevated fetal hemoglobin (HbF) levels are associated with the persistence of splenomegaly [10]. Co-inheritance of α-thalassemia may also contribute to preserving splenic size and function [10]. Furthermore, the spleen responds to SCD by alterations in dimensions and parenchymal structure [5]. Ultrasound imaging can reveal early signs of SCD complications, including splenomegaly, hyposplenism, or splenic infarctions [13].

Existing reports indicate regional variations in parenchymal echotexture and splenic dimensions among SCD patients [14]. However, to our knowledge, no studies have specifically addressed this issue in our local area. Consequently, our study aimed to investigate the dimensions and parenchymal echotexture of the spleen in SCD patients visiting Basra Hematology Center. Additionally, we explored the potential associations between spleen size and variables such as age, type of SCD, lab values, and different genotypes of SCD.

## Materials and methods

Study design and approval

This descriptive cross-sectional study was carried out at Basra Hematology Center in Basra city over a three-month period from May 22 to August 22, 2024. The study sessions were held two days a week, each lasting five hours. The study was approved by the research committee of Basra Health Directorate (approval number 581; May 19, 2024).

Sampling and sample size

A total of 81 patients aged 15 and above, diagnosed with SCD, and attending the outpatient clinic at Basra Hematology Center in Basra city were included. Participants were identified through medical records and referrals from private outpatient clinics.

Data collection procedure

Participants were identified by the authors, approached, and informed about the study's objectives and procedures. Each patient was interviewed in a private setting at the center, using a questionnaire designed to meet the study's objectives. Patients were then scheduled for an ultrasound scan to assess splenic manifestations. Laboratory data was also collected to analyze correlations between ultrasound findings and key hematological parameters.

Interviews

Once patients consented to participate, they underwent an interview during which they completed a standardized questionnaire in a private hospital setting. The interviews lasted approximately 10-15 minutes and aimed to gather comprehensive information about the patient's medical history.

Questionnaire

**Figure 1:**
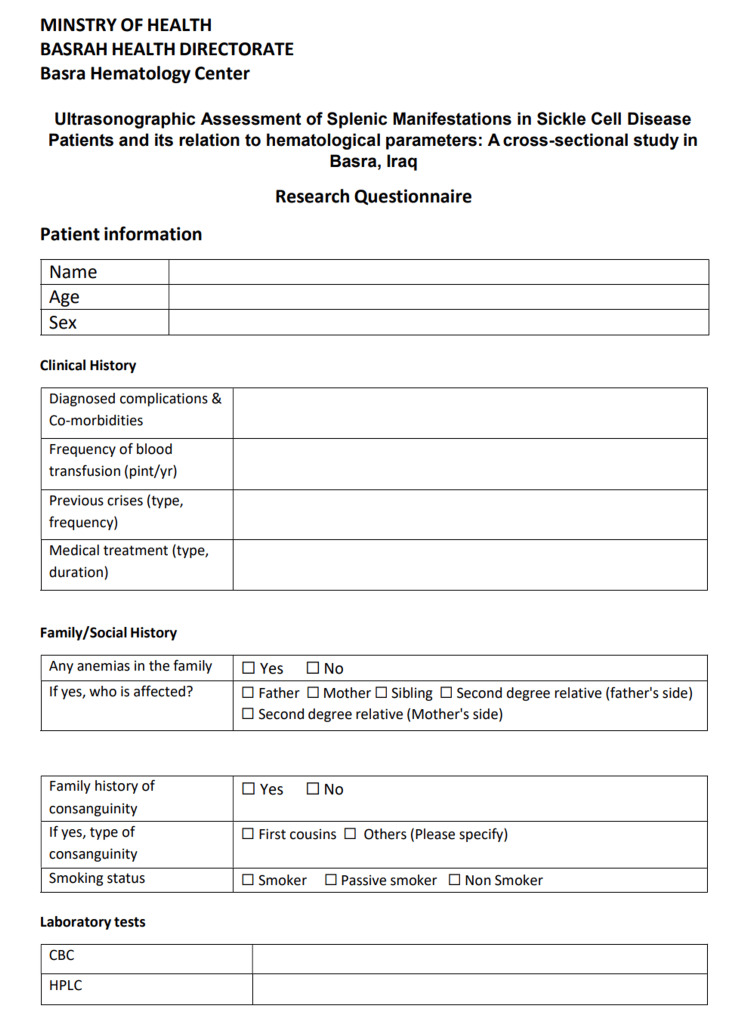
Research questionnaire CBC: Complete blood count; HPLC: High-performance liquid chromatography

The questionnaire included sections on patient demographics, clinical history, family and social history, and previous imaging studies. This structured format ensured a thorough collection of information, supporting the study's objectives. The detailed questionnaire is given in Figure 1 in the Appendices.

Ultrasound and blood samples 

A GE LOGIQ F6 real-time ultrasound machine (GE Medical Systems, China) was used in the study. The device operates with multiple probe frequencies and was calibrated for optimal performance by the technical team before the study. Mild splenomegaly is defined as one standard deviation (SD) above the average spleen size in a healthy population, while massive splenomegaly is two SDs above it. Moderate splenomegaly falls between these two thresholds [5]. A small spleen is defined as having a length ≤7 cm and a width ≤3 cm [15]. Autosplenectomy is described as non-visualization of the spleen in the absence of surgical splenectomy [16].

Blood samples were collected from patients on the day of study recruitment and analyzed at the Basra Hematology Center laboratory. The analysis included parameters such as Hb, white blood cell count (WBC), and platelet count (PLT). The percentages of HbF, HbS, hemoglobin A (HbA), and hemoglobin A2 (HbA2) were determined using high-performance liquid chromatography (HPLC).

The ultrasound examinations were done by two radiology specialists, who performed two separate examinations for each patient. The radiologists were blinded to each other's findings to reduce bias in their observations. The results were compared together while taking the average measurements.

Grouping of subjects

Participants were divided into six groups based on their ultrasound results and spleen size, as previously described in detail: mild splenomegaly, moderate splenomegaly, massive splenomegaly, normal spleen size, small spleen, and autosplenectomy. These groups allowed for the analysis of different splenic manifestations in SCD patients.

Data management

The research team securely stored and managed completed questionnaires, laboratory results, and ultrasound scan reports. Data was stored in both physical and digital formats. Physical copies were accessible only to authorized personnel, while digital data was stored on servers with restricted access.

Ethical considerations

Consent was obtained from each participant prior to their involvement. Each participant received comprehensive information about the study, including their rights, the methods of data collection, and the confidentiality protocols for their data.

Personal information and responses from participants were treated with strict confidentiality and safeguarded against unauthorized access. Data was securely stored and accessible only to authorized researchers.

Inclusion criteria

The study included patients diagnosed with SCD including SCA (HbS/S) and sickle cell-β thalassemia (HbS-β Th), aged 15 and older, of both sexes, who attended the outpatient clinic at Basra Hematology Center.

Exclusion criteria

This study excluded patients under the age of 15 years, patients with only sickle cell trait (HbAS), hemoglobin H disease, patients with other benign or malignant hematological disorders that might affect splenic function, patients with severe co-morbid conditions that might interfere with the study, including advanced hepatic disease and active malignancy, and patients who refused to participate in the study.

Statistical analysis

IBM SPSS Statistics for Windows, Version 29.0.2.0 (IBM Corp., Armonk, US) was used to conduct statistical analysis of the data. The results were organized into tables. The chi-square test (χ²) or Fisher's exact test was utilized to identify relationships among variables. Patients' groups were compared using the one-way analysis of variance (ANOVA) test. Statistical significance was determined with a p-value below 0.05.

## Results

Table 1 shows the demographic characteristics of patients categorized by spleen size and functionality. Age showed a statistically significant difference across the groups (p=0.017), suggesting that marked splenomegaly is associated with younger individuals (mean age of 19.50 ± 4.95 years) compared to autosplenectomy, which is linked to older patients (mean age of 35.42 ± 11.84 years). Gender distribution, however, did not differ significantly, suggesting that both males and females are similarly affected by different spleen statuses (p=0.879). Consanguinity patterns did not display a significant variation, although patients with mild or moderate splenomegaly had a higher percentage of parental consanguinity, particularly among those whose parents were first cousins. Smoking habits showed some variation, with active smoking being more prevalent in the small spleen group; specifically, 50% of participants with a small spleen (four out of eight) were active smokers. However, this difference was not statistically significant (p=0.204). Overall, age appears to be the only demographic factor that significantly correlates with spleen status.

**Table 1:** Demographical data distribution among the studied patients The data are presented as n (%). One-way analysis of variance (ANOVA) test was used to compare the means of age across the different spleen size categories. The chi-square test was used to compare the rest of the variables. Statistical significance was determined with a p-value below 0.05. SD: Standard deviation

variables		Normal spleen	Mild splenomegaly	Moderate splenomegaly	Marked splenomegaly	Small spleen	Autosplenectomy	Statistical test	p-value
Age (years) (Mean ± SD)		24.06 ± 8.59	25.00 ± 10.02	28.93 ± 12.92	19.50 ± 4.95	28.63 ± 8.62	35.42 ± 11.84	F = 5.84	0.017
Gender	Male	8 (47.1%)	7 (35.0%)	8 (53.3%)	1 (50.0%)	4 (50.0%)	7 (36.8%)	Chi-square = 4.4	0.879
	Female	9 (52.9%)	13 (65.0%)	7 (46.7%)	1 (50.0%)	4 (50.0%)	12 (63.2%)		
Consanguinity	No	6 (35.3%)	5 (25.0%)	3 (20.0%)	0 (0.0%)	4 (50.0%)	6 (31.6%)	Chi-square = 10.65	0.447
	1^st^ cousins	9 (52.9%)	13 (65.0%)	9 (60.0%)	1 (50.0%)	4 (50.0%)	13 (68.4%)		
	2^nd^ cousins	2 (11.8%)	2 (10.0%)	3 (20.0%)	1 (50.0%)	0 (0.0%)	0 (0.0%)		
Smoking	No	11 (64.7%)	13 (65.0%)	14 (93.3%)	1 (50.0%)	3 (37.5%)	12 (63.2%)	Chi-square = 17.45	0.204
	Passive	2 (11.8%)	4 (20.0%)	0 (0.0%)	1 (50.0%)	1 (12.5%)	4 (21.1%)		
	Active	3 (17.6%)	3 (15.0%)	1 (6.7%)	0 (0.0%)	4 (50.0%)	2 (10.5%)		
	Ex-smoker	1 (5.9%)	0 (0.0%)	0 (0.0%)	0 (0.0%)	0 (0.0%)	1 (5.3%)		

Table 2 details the medical history and clinical features of the patients, showing no statistically significant differences in the distribution of sickle cell crisis types across spleen status. Acute painful crisis (APC) was universally present across all spleen categories, while severe sickle cell crisis, such as hemolytic crisis, were more frequently observed in patients with autosplenectomy (three out of 19 or 15.8%) and small spleens (one out of 19 or 12.5%), though this trend was not statistically significant (p=0.109). Medical comorbidities, such as hypertension and gallstones, also lacked statistically significant associations, though the autosplenectomy group had a higher frequency of these conditions. Blood transfusion frequency did not vary significantly between the groups (p=0.471), indicating that transfusion requirements were relatively similar regardless of spleen status.

**Table 2:** Medical data distribution among the studied patients The data are presented as n (%). All variables in the table were compared using the chi-square test. Statistical significance was determined with a p-value below 0.05. APC: Acute painful crisis; SSC: Splenic sequestration crisis; AC: Aplastic crisis; HC: Hemolytic crisis; HTN: Hypertension; DM: Diabetes mellitus; AVN: Avascular necrosis

variables		Normal spleen	Mild splenomegaly	Moderate splenomegaly	Marked splenomegaly	Small spleen	Autosplenectomy	Chi-square value	p-value
Sickle cell crisis type	APC	17 (100%)	20 (100%)	15 (100%)	2 (100%)	8 (100%)	19 (100%)	-	-
	SSC	0 (0.0%)	1 (5.0%)	3 (20.0%)	1 (50.0%)	0 (0.0%)	2 (10.5%)	9.57	0.094
	ACS	0 (0.0%)	1 (5.0%)	1 (6.7%)	0 (0.0%)	0 (0.0%)	1 (5.3%)	1.63	0.924
	aplastic	1 (5.9%)	0 (0.0%)	0 (0.0%)	0 (0.0%)	0 (0.0%)	1 (5.3%)	2.58	0.778
	Hemolytic	0 (0.0%)	0 (0.0%)	0 (0.0%)	0 (0.0%)	1 (12.5%)	3 (15.8%)	8.55	0.109
Medical history	HTN	0 (0.0%)	1 (5.0%)	1 (6.7%)	0 (0.0%)	0 (0.0%)	3 (15.8%)	4.74	0.516
	DM	0 (0.0%)	0 (0.0%)	0 (0.0%)	0 (0.0%)	0 (0.0%)	1 (5.3%)	3.25	0.750
	Gallstones	2 (11.8%)	3 (15.0%)	1 (6.7%)	0 (0.0%)	2 (25.0%)	5 (26.3%)	3.57	0.652
	AVN	6 (35.5%)	3 (15.0%)	3 (20.0%)	0 (0.0%)	4 (50.0%)	8 (42.1%)	6.83	0.248
	Stroke	1 (5.9%)	0 (0.0%)	0 (0.0%)	0 (0.0%)	0 (0.0%)	2 (10.5%)	4.44	0.585
Blood transfusion (pint/year)	No	14 (82.4%)	15 (75.0%)	10 (66.7%)	1 (50.0%)	5 (62.5%)	14 (73.7%)	26.45	0.471
	1	0 (0.0%)	1 (5.0%)	2 (13.3%)	0 (0.0%)	2 (25.0%)	1 (5.3%)		
	2	1 (5.9%)	0 (0.0%)	1 (6.7%)	1 (50.0%)	1 (12.5%)	0 (0.0%)		
	3	1 (5.9%)	2 (10.0%)	1 (6.7%)	0 (0.0%)	0 (0.0%)	3 (15.8%)		
	4	0 (0.0%)	0 (0.0%)	1 (6.7%)	0 (0.0%)	0 (0.0%)	0 (0.0%)		
	≥5	1 (5.9%)	2 (10.0%)	0 (0.0%)	0 (0.0%)	0 (0.0%)	1 (5.3%)		
APC frequency per year	<5	11 (64.71%)	9 (45.0%)	9 (60.0%)	1 (50.0%)	5 (62.5%)	10 (55.6%)	8.21	0.942
	5-10	3 (17.65%)	6 (30.0%)	5 (33.3%)	1 (50.0%)	2 (25.0%)	5 (27.8%)		
	>10	3 (17.65%)	5 (25.0%)	1 (6.7%)	0 (0.0%)	1 (12.5%)	3 (16.7%)		

Table 3 highlights significant differences in radiological findings related to spleen size and morphology. Splenic length, width, and thickness were all significantly different across groups (p<0.001), with normal spleens having the smallest dimensions and marked splenomegaly showing the largest. These differences were expected given the categorization by spleen size. Echogenicity showed a marked contrast, where increased echogenicity was more frequently seen in the small spleen group (seven out of eight or 87.5%, p<0.001). Focal lesions, such as infarctions, were relatively uncommon and did not show significant variation across the groups. However, the presence of GGBs was reported in a small number of patients and did not vary significantly among spleen status groups.

**Table 3:** Radiological data distribution among the studied patients The data are presented as mean ± SD for the splenic length, splenic width, and splenic thickness rows. The data for the remaining rows is presented as n(%). One-way analysis of variance (ANOVA) test was used to compare the means of splenic length, width, and thickness between the spleen size categories. The chi-square test was used to compare the rest of the variables. Statistical significance was determined with a p-value below 0.05. SD: Standard deviation

Variables		Normal spleen	Mild splenomegaly	Moderate splenomegaly	Marked splenomegaly	Small spleen	Autosplenectomy	Statistical test	p-value
Splenic length (cm) (Mean ± SD)		10.03 ± 4.1	15.07 ± 0.75	17.73 ± 0.95	20.15 ± 0.49	5.46 ± 1.57	0.0	F-value = 1188.92	<0.001
Splenic width (cm) (Mean ± SD)		7.12 ± 3.12	10.38 ± 1.34	12.53 ± 1.71	13.10 ± 0.14	4.33 ± 1.36	0.0	F-value = 115.58	<0.001
Splenic thickness (cm) (Mean ± SD)		3.59 ± 1.74	5.89 ± 0.99	7.05 ± 0.88	5.95 ± 0.49	2.54 ± 1.50	0.0	F-value = 67.18	<0.001
Echogenecity	Normal	13 (76.5%)	18 (90.0%)	14 (93.3%)	2 (100.0%)	1 (12.5%)	0 (0.0%)	Chi-square = 112.18	<0.001
	increased	4 (23.5%)	2 (10.0%)	1 (6.7%)	0 (0.0%)	7 (87.5%)	0 (0.0%)		
Focal lesions	No focal lesion	12 (70.6%)	14 (70.0%)	11 (73.3%)	2 (100.0%)	7 (87.5%)	19 (100.0%)	Chi-square = 12.87	0.185
	Infarction	4 (23.5%)	4 (20.0%)	1 (6.7%)	0 (0.0%)	1 (12.5%)	0 (0.0%)		
	Gamna-Gandy bodies	1 (5.9%)	2 (10.0%)	3 (20.0%)	0 (0.0%)	0 (0.0%)	0 (0.0%)		

The laboratory data showed notable significant differences in several key blood parameters (Table 4). Hb levels were significantly lower in patients with marked splenomegaly (6.65 ± 0.21 g/dl) compared to other groups (p=0.01), reflecting more severe anemia in these patients likely due to increased hemolysis. WBC and PLT levels were significantly higher in the autosplenectomy group (WBC 12.29 ± 5.67 x 10³/μL; PLT 426.16 ± 222.85 x 10³/μL), both with p-values <0.001. HbS% also differed significantly (p=0.008), with the highest levels seen in patients with marked splenomegaly and autosplenectomy, which may indicate more severe sickling in these groups. Other parameters, such as HbA%, HbA2%, and HbF%, did not show significant differences, though HbF% was borderline (p=0.063), which may reflect HbF's role in disease modulation.

**Table 4:** Laboratory data distribution among the studied patients The data are presented as mean ± SD. One-way analysis of variance (ANOVA) test was used to compare the means of laboratory parameters across the six spleen size categories. Statistical significance was determined with a p-value below 0.05. Hb: Hemoglobin; WBC: White blood cell count; PLT: Platelet count; HbA: Hemoglobin A; HbA2: Hemoglobin A2; HbF: Hemoglobin F; HbS: Hemoglobin S; SD: Standard deviation

Variables	Normal spleen	Mild splenomegaly	Moderate splenomegaly	Marked splenomegaly	Small spleen	Autosplenectomy	F-value	p-value
Hb g/dl (Mean ± SD)	10.16 ± 1.83	9.73 ± 2.35	9.47 ± 2.08	6.65 ± 0.21	9.58 ± 1.46	8.07 ± 1.61	1.96	0.01
WBC 10^3^ /μL (Mean ± SD)	9.64 ± 4.97	6.94 ± 2.73	6.83 ± 2.61	7.33 ± 2.08	10.09 ± 2.74	12.29 ± 5.67	2.71	<0.001
PLT 10^3^ /μL (Mean ± SD)	390.82 ± 195.44	227.50 ± 104.11	201.93 ± 179.63	254.50 ± 177.48	454.00 ± 102.51	426.16 ± 222.85	5.49	<0.001
HbA% (Mean ± SD)	5.75 ± 7.75	5.73 ± 7.68	7.31 ± 17.78	3.75 ± 5.30	3.79 ± 4.82	6.99 ± 8.84	0.93	0.674
HbA2% (Mean ± SD)	3.28 ± 1.91	3.77 ± 2.05	3.81 ± 1.45	4.65 ± 3.61	3.76 ± 2.21	3.65 ± 1.26	1.24	0.908
HbF% (Mean ± SD)	17.66 ± 7.07	21.74 ± 14.45	16.95 ± 8.39	11.70 ± 7.78	12.63 ± 6.45	12.05 ± 6.84	2.78	0.063
HbS% (Mean ± SD)	69.78 ± 9.42	67.44 ± 11.69	70.18 ± 15.44	79.90 ± 16.69	77.38 ± 9.50	78.53 ± 11.28	4.24	0.008

Table 5 aimed to assess whether the Hb type (specifically HbS/S and HbS-β Th) influences spleen size in SCD patients. The results showed that the distribution of these genotypes across different spleen size categories was not significantly different. Both HbS/S and HbS-β Th hemoglobin types were represented similarly across all categories, with p-values of 0.916, indicating no statistically significant association between Hb type and spleen size.

**Table 5:** Hemoglobin type distribution among the studied patients The data are presented as n (%). The distribution of hemoglobin types across spleen size categories was compared using the chi-square test. Statistical significance was determined with a p-value below 0.05. HbS/S: Sickle cell anemia; HbS-β Th: Sickle cell-β thalassemia

variables		Normal spleen	Mild splenomegaly	Moderate splenomegaly	Marked splenomegaly	Small spleen	Autosplenectomy	Chi-square value	p-value
Hemoglobin type	HbS/S	7 (41.2%)	7 (35.0%)	7 (46.7%)	1 (50.0%)	4 (50.0%)	6 (31.6%)	1.47	0.916
	HbS/β Th	10 (58.8%)	13 (65.0%)	8 (53.3%)	1 (50.0%)	4 (50.0%)	13 (68.4%)		

## Discussion

Our study looked at the correlation of spleen status assessed by ultrasound with the radiological, clinical, and laboratory findings in patients with SCD. Our findings were consistent with the pathophysiology of SCD as described in the literature, particularly with regard to the relationship between splenic size and age and the presence of changes in splenic parenchyma and focal splenic changes, including infarctions and GGBs. Key hematological parameters, such as Hb levels, WBC, and PLT, showed significant variation across different spleen size categories, reflecting the connection between splenic function and the various blood cells in SCD patients.

Splenic size and age association

Our study showed that autosplenectomy is more common in older patients, in contrast to splenomegaly, which has a higher prevalence in younger age groups. These observations are similar to those of other studies, such as those by Ugwu et al. [17] and Ladu et al. [18], which presented a progressive reduction in splenic size with advancing age. This is likely due to repeated vaso-occlusive crises and resultant ischemic damage to the spleen. However, a percentage of our study population was found to have a normal spleen size. This is in contrast to the conventional belief that most patients have autosplenectomy by the age of five years [10]; factors that may have led to this may include a higher level of HbF or treatment with HbF-inducing treatments such as hydroxyurea.

We noticed a higher prevalence of consanguinity among patients with splenomegaly. This may influence the occurrence of splenomegaly in SCD patients by increasing the prevalence of shared genetic traits that exacerbate disease severity. These findings correlate with studies from other regions with high consanguinity rates, such as Saudi Arabia [13], which also reported similar associations between familial genetic patterns and the state of the spleen.

Echogenicity and parenchymal changes

We identified increased parenchymal echogenicity in patients with small spleens, suggesting fibrosis or other pathological changes. This finding is consistent with a study done in a teaching hospital in Nigeria by Eze et al. [5], which reported the presence of hyperechoic and fibrotic parenchymal texture of the spleen among patients whose spleen has persisted into adulthood. Splenic infarctions were found in about 12% (10 out of 81) of the patients in our study, with no significant association with splenic size. Eze et al. also reported a close percentage, most likely due to a similar study structure examining relatively stable patients visiting the outpatient clinic. GGBs were found in a small proportion of our patients. Previous studies, including one conducted at Lady's Children's Hospital, Dublin [12], also noted this feature. The changes mentioned above support ultrasound as a reliable modality for detecting chronic sequelae of SCD.

Hematological parameters and disease severity

Regarding the lab values, we found a significant association between low Hb levels and marked splenomegaly. This aligns with what is mentioned in a review article by Wilson-Okoh et al. [19], which states that hemolytic anemia is responsible for increased splenic size. Splenomegaly primarily results from the spleen’s intricate structure, which promotes stasis, anoxia, and the trapping of sickle erythrocytes within the splenic pulp and sinuses [19]. This shows that although some patients might be protected against vaso-occlusion and the subsequent reduction in splenic size, they are still susceptible to hemolysis and splenomegaly.

The relationship between WBC and PLT levels with the severity of SCD was previously explored by Helvaci et al., who found that PLT and, to a less significant extent, WBC are associated with a more aggressive disease course characterized by frequent crises [20]. This is probably due to an inflammatory response that occurs due to the SCD activity. This can explain why we found a significant association between high WBC and PLT levels and the presence of autosplenectomy, as these patients tend to have high levels of HbS, which correspond to more disease severity and, subsequently, more inflammation. The inflammation in SCD results from multiple complex mechanisms that cause the release of inflammatory markers, including IL-6, TNFα, and IL-1β in monocytes [21].

Concerning HbF, although the p-value is borderline (p=0.063), several studies mentioned that higher levels of HbF could provide some protection against autosplenectomy and small spleen size. The presence of high HbF compensates for the hypoxic episodes and reduces the severity of sickling, which in turn lowers the risk of splenic infarction and helps preserve splenic function [22].

Regarding Hb types, although patients with HbS-β Th had a higher incidence of splenomegaly and less incidence of autosplenectomy and small-sized spleen, this finding was not statistically significant in our study. This contrasts with the existing literature, which generally associates splenomegaly with heterozygous forms of SCD, such as sickle-hemoglobin C (HbS/C) and HbS-β Th [23]. A study in Jamaica [23] compared HbS-β Th with homozygous SCD and found that splenomegaly is more commonly persistent in patients with HbS-β Th. They attributed their findings to the reduced intravascular sickling in these heterozygotes, likely due to a lower mean corpuscular concentration of HbS. Another factor is the HbF levels, which are typically lower in individuals with HbS/S, leading to more severe sickling and increased complications such as autosplenectomy and small spleen [24]. In contrast, in sickle β-thalassemia, genetic factors and modifiers may upregulate HbF production, reducing sickle-related complications [25].

There are several potential explanations for why these findings may differ from the existing literature. The small sample size may have influenced our results. Moreover, some of the SS homozygotes with splenomegaly may be, in fact, heterozygotes, such as patients with the α-thalassemia genotype, where splenomegaly is more prevalent [26,27]. The α component can be difficult to detect with standard HPLC used in this study due to the low abundance and weak chromatographic separation of these components. Additionally, it has low sensitivity for some α-globin variants [28]. Another factor is reduced sickling by the use of hydroxyurea, which induces HbF synthesis [29]. These factors could have affected our results and led to this lack of significance.

Study limitations

Although our study provides valuable insights, some limitations are present and must be acknowledged. Firstly, the cross-sectional design limits the ability to establish causal relationships between our studied parameters over time. Hence, conducting longitudinal studies to monitor splenic changes and their correlation with our examined parameters would provide a deeper insight into the progression of splenic involvement in SCD. Secondly, while one of the aims of our study was to explore the regional genetic or environmental factors that might affect SCD patients in our region of Basra, Iraq, said changes might limit the generalizability of our findings to other populations. Furthermore, our sample is relatively small compared to the burden of SCD. Future studies should involve a larger sample and the use of genetic testing to accurately evaluate the genotype of the participants. Moreover, Exploring potential factors that could limit splenic damage, such as hydroxyurea or other SCD treatments, can also provide more insight into how to prevent such damage. Additionally, adding more imaging studies, such as CT scans or MRI, and comparing them to the sonographic assessment can add further value to the results and reduce the interobserver variability of ultrasound scanning.

## Conclusions

This study examined splenic changes in SCD patients as detected by ultrasound assessment and how these findings relate to various hematological parameters. We found that autosplenectomy is more common in older patients, while splenomegaly is prevalent in younger ones. Increased splenic echogenicity had a significant association with small-sized spleens, indicating parenchymal fibrosis in such patients. Focal splenic changes such as splenic infarctions and GGBs are reported in a small proportion of patients with SCD. Patients with splenomegaly showed lower Hb levels, while patients with autosplenectomy showed higher WBC and PLT levels. The findings emphasize the importance of closely monitoring older SCD patients for splenic atrophy and considering earlier interventions. Routine screening for splenic function and proactive management of Hb levels may improve outcomes, though the protective role of elevated HbF and genetic factors requires further investigation due to a lack of statistical significance.
